# The treatment of selected traditional Chinese medicine prescriptions and their metabolites for heart failure with preserved ejection fraction from basic science discovery to the clinic

**DOI:** 10.3389/fphar.2026.1927962

**Published:** 2026-09-15

**Authors:** Yanhong Zhang, Wanqing Wu, Xi Zhai, Jingkai Zhang, Weidong Wang, Xinyuan Huang, Lan Wu

**Affiliations:** 1 Graduate School, Shanghai University of Traditional Chinese Medicine, Shanghai, China; 2 Shanghai Key Laboratory of Molecular Imaging, Zhoupu Hospital, Shanghai University of Medicine and Health Sciences, Shanghai, China; 3 Institute of Wound Prevention and Treatment, Shanghai University of Medicine and Health Sciences, Shanghai, China; 4 School of Basic Medical Science, Shanghai University of Medicine and Health Sciences, Shanghai, China; 5 School of Health Science and Engineering, University of Shanghai for Science and Technology, Shanghai, China; 6 School of Clinical Medical Science, Shanghai University of Medicine and Health Sciences, Shanghai, China; 7 Shanghai University of Traditional Chinese Medicine, Shanghai, China

**Keywords:** guideline-directed medical therapy, heart failure with preserved ejection fraction, pathophysiological mechanisms, traditional Chinese medicine, treatment

## Abstract

The prevalence of heart failure with preserved ejection fraction (HFpEF) continues to rise worldwide. Compared with heart failure with reduced ejection fraction, HFpEF is associated with significant morbidity and mortality, yet only a limited number of treatment options have been proven effective. This narrative review aims to summarize the pathophysiological mechanisms of HFpEF and the pharmacological therapeutic strategies for this condition. Through a search of relevant literature from PubMed, VIP, Wanfang Data, and CNKI, basic research and clinical data on HFpEF were collected. The process involved examining and analyzing research on mechanisms and treatments of HFpEF. Based on both basic research and clinical data, this paper discussed the key roles of anti-inflammatory effects, anti-oxidant effects, and the reversal of mitochondrial dysfunction in improving cardiac remodeling, cardiac dysfunction and exercise intolerance in HFpEF. Furthermore, the paper suggested that Traditional Chinese Medicine (TCM), including metabolites and prescriptions, may serve as a potential therapeutic strategy for HFpEF and some prescriptions may serve as a potential adjuvant to guideline-directed medical therapy. In the future, it is hoped that the prognosis for patients with HFpEF by promoting the usage of TCM can be improved.

## 1 Introduction

Heart failure (HF) is the leading cause of death among cardiovascular diseases. It manifests as a complex syndrome rather than a single pathological diagnosis in clinic. The characteristic features of this clinical syndrome include typical symptoms such as dyspnea, ankle swelling, and persistent fatigue, along with physical signs such as elevated jugular venous pressure, pulmonary crackles, and peripheral edema. From a pathophysiological perspective, patients with HF typically exhibit significant abnormalities in cardiac structure and function (Bozkurt et al, 2021).

Modern cardiology has moved beyond the simple concept of “heart pump failure” and now views HF as a complex pathological process. According to the latest standards of the 2021 European Society of Cardiology (ESC) guidelines (Mcdonagh et al, 2021) and the 2022 American College of Cardiology/American Heart Association (ACC/AHA) guidelines (Heidenreich et al, 2022), HF was classified into four main types based on the key indicator of left ventricular ejection fraction (LVEF): Heart failure with reduced ejection fraction (HFrEF): Characterized by typical heart failure symptoms and an LVEF ≤ 40%. Heart failure with mildly reduced ejection fraction (HFmrEF): Patients exhibit clear symptoms of heart failure, with an LVEF between 41% and 49% Heart failure with preserved ejection fraction (HFpEF): Patients exhibit typical clinical symptoms of heart failure, with an LVEF ≥ 50%. Heart failure with improved ejection fraction (HFimpEF): This specifically refers to patients with a baseline LVEF ≤ 40%, after treatment, experience an increase in LVEF of ≥10 percentage points, with a second measurement showing LVEF > 40%.

Notably, with the acceleration of population aging and the rising prevalence of metabolic diseases, the disease burden of HFpEF is showing a sustained upward trend globally. This shift poses new challenges to public health systems and clinical practice. This review focused on the clinic and basic research of HFpEF and its treatment. It summarized the pharmacotherapy for HFpEF, including guideline-directed medical therapy (GDMT) and traditional Chinese medicine (TCM). It will provide new insight into TCM in the treatment of HFpEF.

## 2 HFpEF requires attention

### 2.1 Clinical manifestation of HFpEF

HFpEF accounts for over 50% of all HF patients. The incidence of this disease is significantly higher in the elderly population and exhibits marked gender differences—the incidence rate is higher in women than in men, particularly in elderly women with long-standing hypertension (Bhuiyan and Maurer, 2011; Shiga et al, 2019; Wang et al, 2021). Population-based and clinical registration studies indicate that HFpEF patients often have multiple phenotypes, including obesity, coronary artery disease, diabetes, atrial fibrillation (AF), hypertension and dyslipidemia (Chioncel et al, 2017; Ge, 2020; Ma et al, 2020; Shah et al, 2017).

Due to the relatively normal LVEF retained by patients with HFpEF, the diagnostic process presents numerous challenges. HFpEF was often referred to as “diastolic heart failure” in early literature, and its pathophysiological characteristics differ from those of traditional systolic heart failure, requiring comprehensive evidence from clinical manifestations, imaging studies, and biomarkers (Chioncelet al., 2017). The diagnosis of HFpEF requires the key criteria showed in Table 1.

**TABLE 1:** Diagnostic standard for HFpEF.

Diagnostic dimension	Key indicators/diagnostic criteria	Interpretation and clinical significance
Natriuretic peptides	Sinus rhythm: NT-proBNP ≥ 125 pg/mL or BNP ≥ 35 pg/mL Atrial fibrillation: NT-proBNP ≥ 375 pg/mL or BNP ≥ 105 pg/mL	Assesses ventricular wall stress and volume overload; thresholds are elevated in AF
Echocardiography	Elevated filling pressure: mean E/e′ > 14, TR peak velocity >2.8 m/s Structural remodeling: LAVI >34 mL/m^2^ (>40 in AF) LVMI > 115 g/m^2^ (males)/> 95 g/m^2^ (females); RWT > 0.42	Provides non-invasive evidence of impaired LV diastolic function and LA/LV structural remodeling
Validated algorithms	H2FPEF score (weighted clinical and echocardiographic variables) HFA-PEFF diagnostic pathway (comprehensive functional and structural scoring)	H2FPEF: score ≥ 6 confirms diagnosis; 2–5 indicates intermediate probability; 0–1 rules out HFA-PEFF: Score ≥ 5 confirms diagnosis; 2–4 warrants advanced stress testing
Exercise/invasive testing	Exercise stress echo: post-exercise mean E/e′ > 14 or TR velocity > 3.4 m/s Right heart catheterization (gold standard): resting PCWP ≥ 15 mmHg (or LVEDP ≥ 16 mmHg) Exercise PCWP ≥ 25 mmHg (or ΔPCWP/ΔCO > 2.0 mmHg/L/min)	Evaluates hemodynamic reserve and confirms diagnosis in patients in the resting “gray zone” (intermediate probability)

NT-proBNP, N-terminal B-type brain natriuretic peptide precursor; BNP, B-type brain natriuretic peptide; AF, atrial fibrillation; E/e′, Early left ventricular filling velocity/Early mitral annular diastolic velocity; TR, tricuspid regurgitation; LAVI, left atrial volume index; LVMI, left ventricular mass index; RWT, relative wall thickness; LA, left atrial; LV, left ventricular; H2FPEF, Heavy (BMI > 30), Hypertension, Atrial Fibrillation, Pulmonary Hypertension, Elder, Filling pressure; HFA-PEFF, Heart Failure Association - Preserved Ejection Fraction; PCWP, pulmonary capillary wedge pressure; LVEDP, left ventricular end-diastolic pressure; ΔPCWP/ΔCO, change in pulmonary capillary wedge pressure per change in cardiac output.

### 2.2 Molecular mechanisms of HFpEF

In HFpEF, cardiac remodeling and dysfunction induced by different triggers of interaction involved abnormalities in inflammatory responses (Figure 1), oxidative stress (Figure 2), and mitochondrial dysfunction (Figure 3) that caused cardiomyocyte injury, coronary microvascular endothelial dysfunction, activated immune cells infiltration, and reactive interstitial fibrosis.

**FIGURE 1:**
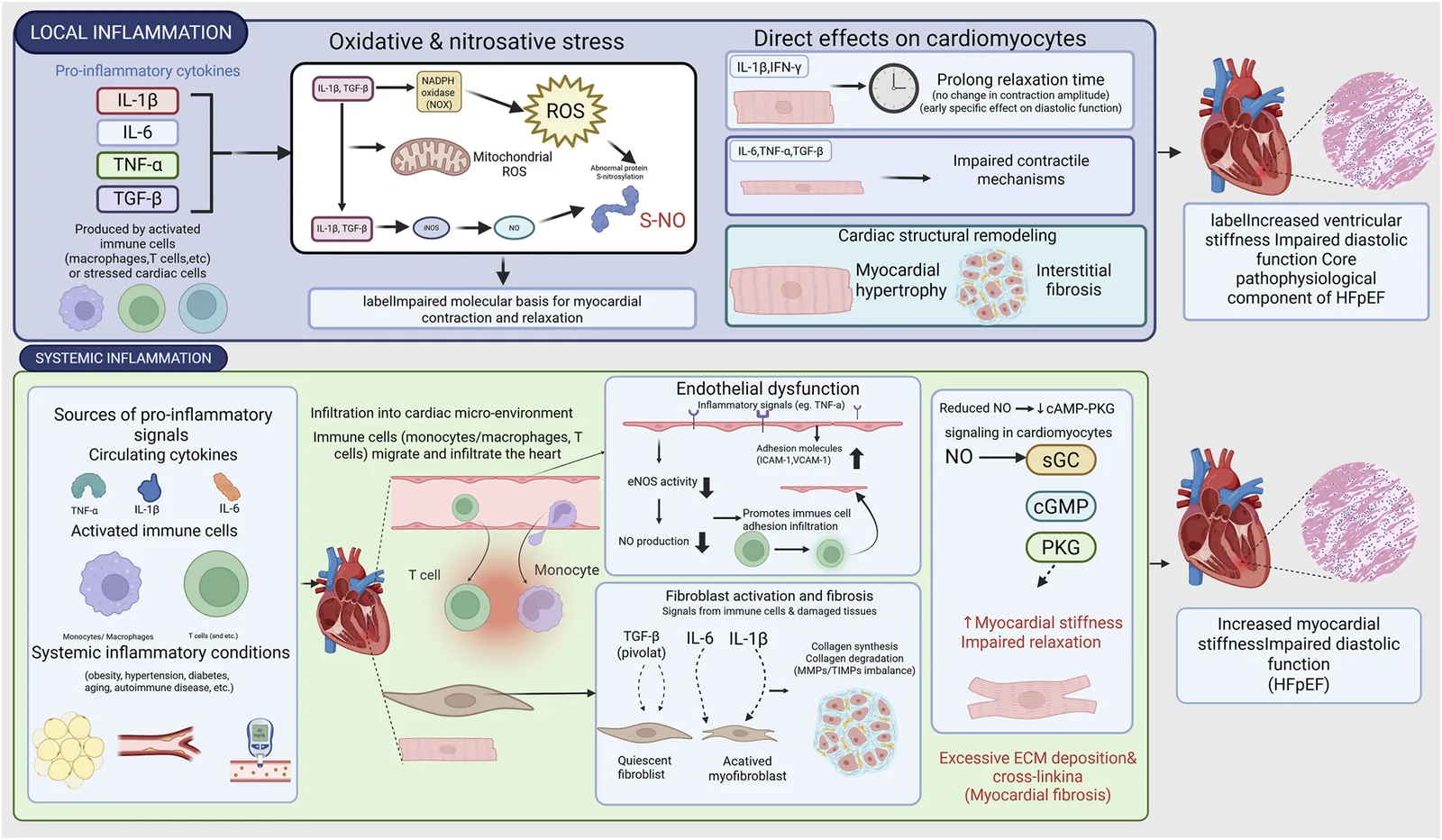
Mechanisms underlying the local and systemic inflammation in HFpEF. IL-1β, Interleukin-1β; IL-6, interleukin-6; TNF-α,Tumor Necrosis Factor-α; TGF-β, transforming growth factor-β; ROS, reactive oxygen species; iNOS, inducible nitric oxide synthase; IFN-γ, Interferon-γ; eNOS, endothelial nitric oxide synthase; ICAM-1, intercellular adhesion molecule-1; VCAM-1, vascular cell adhesion molecule 1; PKG, protein kinase G; MMPs, matrix metalloproteinases; TIMPs, tissue inhibitors of metalloproteinases; ECM, excessive extracellular matrix; MCP-1, monocyte chemoattractant protein-1. Created with BioRender.com.

**FIGURE 2:**
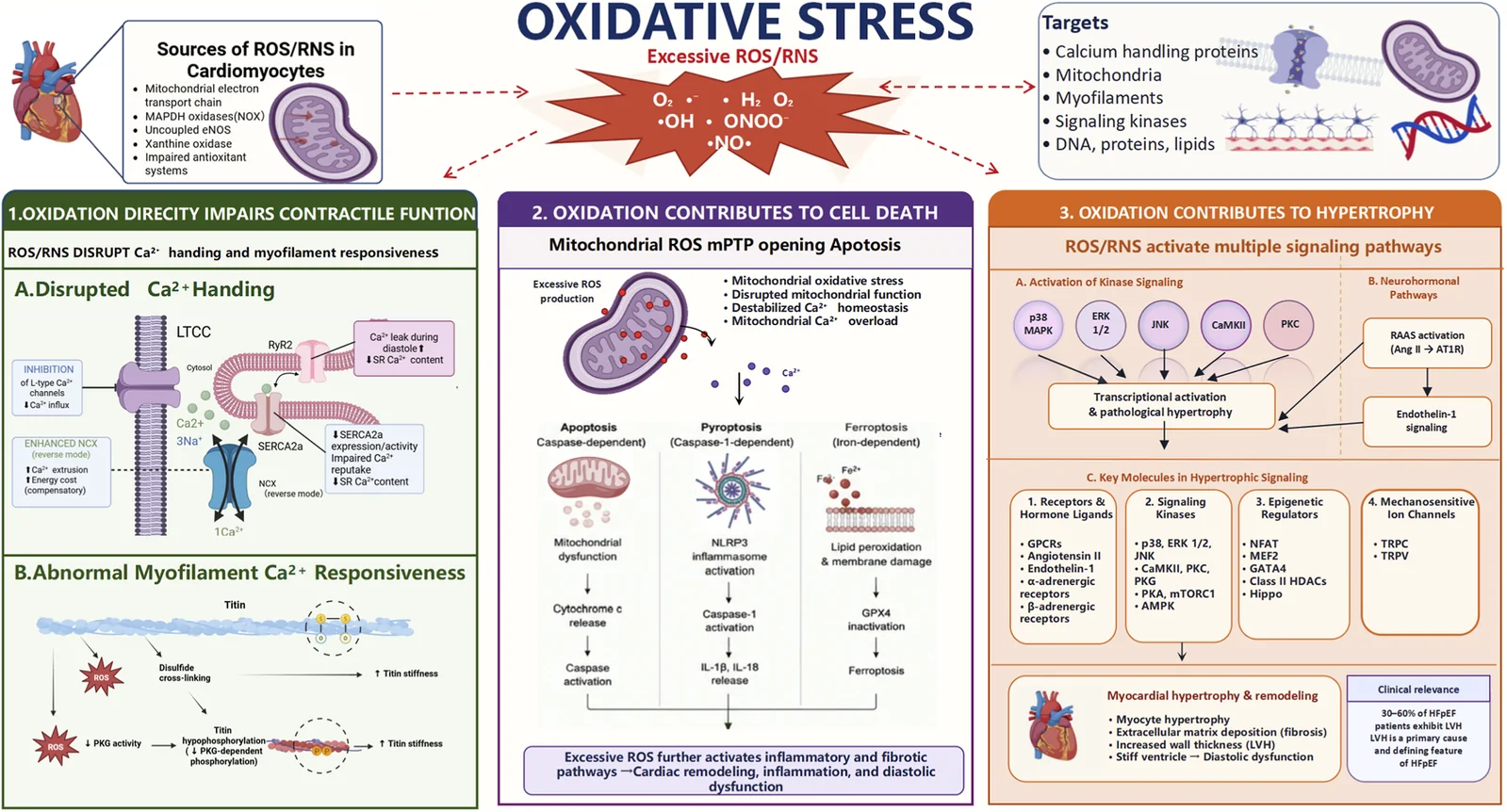
Oxidation stress drives the progression of HFpEF RNS, reactive nitrogen species; SERCA2a, sarcoplasmic/endoplasmic reticulum calcium ATPase 2; RyR2, ryanodine receptor 2; mPTP, mitochondrial permeability transition pore; p38, p38 mitogen-activated protein kinase; ERK1/2, extracellular regulated protein kinases 1/2; JNK, c-Jun N-terminal kinase; CaMKII, calcium/calmodulin-dependent protein kinase II; PKC, protein kinase C; RAAS, renin-angiotensin-aldosterone system; LVH, left ventricular hypertrophy; GPCRs, G protein-coupled receptors; PKA, protein kinase A; mTORC1, mammalian target of rapamycin complex 1; AMPK, AMP-activated protein kinase; NFAT, nuclear factor of activated T-cells; MEF2, myocyte enhancer factor 2; GATA4, GATA binding protein 4; Class II HDACs, class II histone deacetylases; TRPC, transient receptor potential canonical; TRPV, transient receptor potential vanilloid. Created with BioRender.com.

**FIGURE 3:**
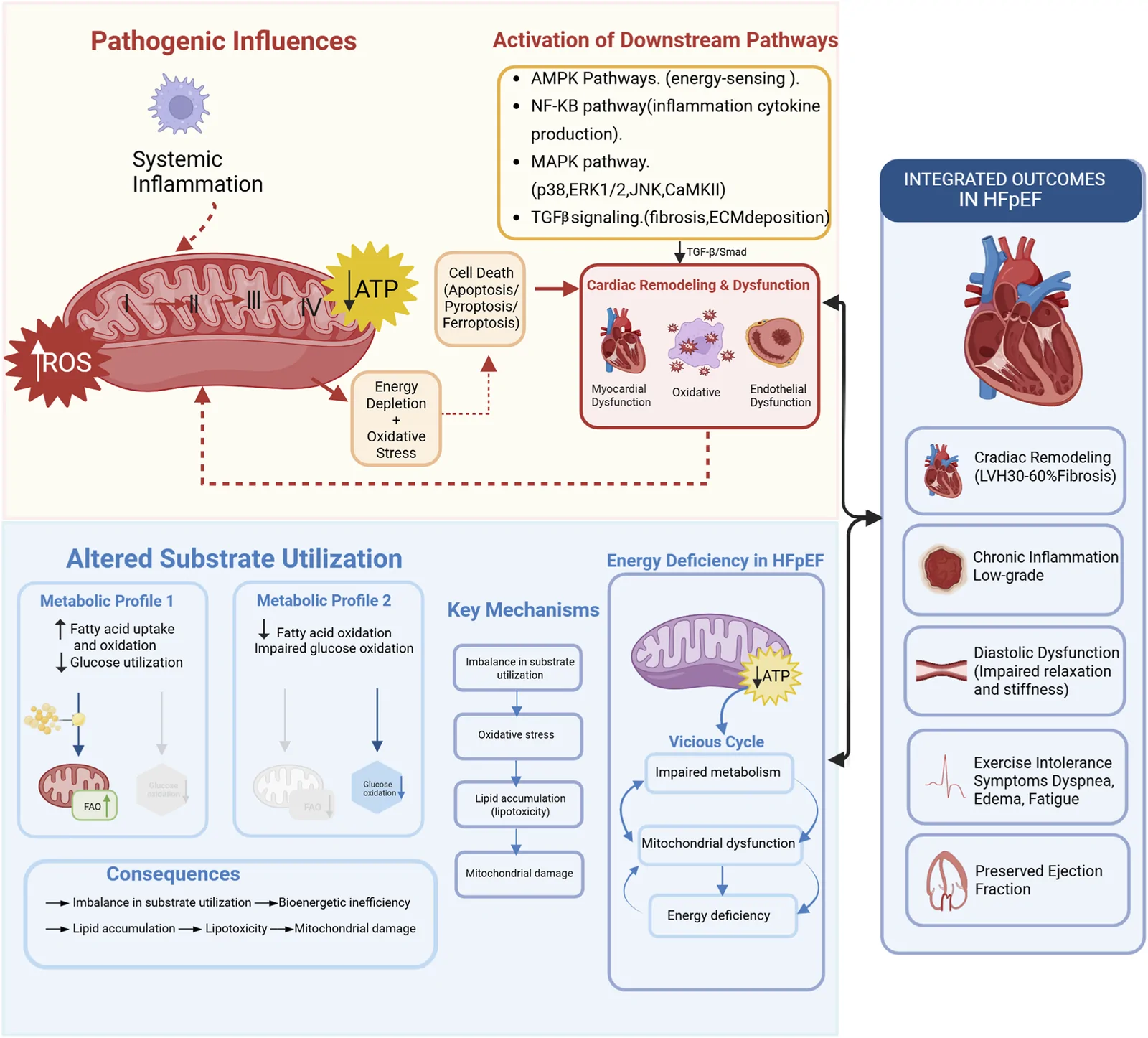
Mitochondrial dysfunction in HFpEF ATP, adenosine triphosphate; NF-κB, nuclear factor κB; MAPK, mitogen-activated protein kinase; mTOR, mammalian target of rapamycin. Created with BioRender.com.

### 2.3 Inflammatory responses

#### 2.3.1 Local inflammation

In HFpEF pathogenesis, pro-inflammatory cytokines (interleukin-1β (IL-1β), interleukin-6 (IL-6), tumor necrosis factor-α (TNF-α), transforming growth factor-β (TGF-β) and Interferon-γ(IFN-γ)) drive cardiac remodeling and dysfunction via distinct cellular pathways (Paulus and Tschöpe, 2013). IL-1β and TGF-β could activate myocardial NADPH oxidase (NOX), induce mitochondrial reactive oxygen species (ROS) production (Ge, 2020; Shah et al, 2017), and promote inducible nitric oxide synthase (iNOS) expression to cause abnormal protein S-nitrosylation, resulting in impaired contractile-relaxation (Chaar et al, 2022; Ma et al., 2020). IL-1β and IFN-γ directly prolonged myocardial cardiomyocyte relaxation time without affecting contraction amplitude, indicating an early selective impact on diastolic function (Westermann et al, 2011). IL-6, TNF-α, and TGF-β not only directly impaired myocardial cell contractile mechanisms but also drove cardiac structural remodeling through myocardial hypertrophy and interstitial fibrosis, ultimately elevating cardiaomyocyte stiffness (Srivaratharajah et al, 2016). These cytokine-driven oxidative, nitrosative, cardiac dysfunction and structural alterations represent a core pathophysiological mechanism in HFpEF.

#### 2.3.2 Systemic inflammation

Systemic inflammation triggers HFpEF pathogenesis through coordinated multi-cellular signal sources: circulating pro-inflammatory cytokines (TNF-α, IL-1β, IL-6) and infiltrating immune cells (monocytes, macrophages, T cells) delivered inflammatory signals to the cardiac microenvironment (Srivaratharajah et al., 2016). Endothelial dysfunction and fibroblast activation in the heart were two main results via the inflammatory signals. TNF-α reduced endothelial nitric oxide synthase (eNOS) activity and nitric oxide (NO) production, impairing cardiomyocyte cGMP/protein kinase G (PKG) signaling and elevating myocardial stiffness (Mcdonagh et al., 2021). Besides, receptor activation on endothelial cells via the inflammatory signals upregulated adheresion molecules, promoting further immune cell infiltration and amplifying local inflammation (Bozkurt et al., 2021). Transmitted signals via TGF-β receptors and IL-1 receptors triggered key intracellular pathways in fibroblasts, altering their function and establishing a chronic inflammatory feedback loop (Bozkurt et al., 2021). Immune-cell-derived TGF-β (synergized by IL-1/IL-6) (Bhuiyan and Maurer, 2011; Heidenreich et al., 2022; Wang et al., 2021) converted fibroblasts into myofibroblasts via activating Smad and it finally caused cardiac fibrosis via collagen deposition (Shiga et al., 2019; Chioncel et al., 2017; Franssen et al, 2016). These interactions directly drove adverse cardiac remodeling and dysfunction.

### 2.4 Oxidative stress

#### 2.4.1 Oxidation directly impairs contractile function

In HFpEF, oxidative stress directly impairs contractile function by post-translationally modifying sarcomeric and excitation-contraction coupling proteins. ROS induced disulfide cross-linking in titin, increasing myocyte passive stiffness. Concurrently, ROS suppressed PKG activity leads to titin hypophosphorylation, which directly elevated cardiomyocyte passive stiffness and impaired myocardial diastolic relaxation (Beckendorf and Linke, 2015). Oxidative inactivation of sarcoplasmic/endoplasmic reticulum calcium ATPase 2 (SERCA2a) activity, phospholamban inhibition, and ryanodine receptor (Ryr) hyperphosphorylation impaired Ca^2+^ handling and sarcoplasmic reticulum (SR) reuptake, worsening myocardium relaxation (Van Heerebeek et al, 2006). Ultimately, these direct oxidative modifications to titin rigidity and calcium transport drove the severe cardiac dysfunction characteristic of HFpEF.

#### 2.4.2 Oxidation contributes to cell death

In HFpEF, oxidative stress triggers multiple regulated cell death pathways that accelerate cardiomyocyte loss and adverse cardiac remodeling. Excessive ROS compromised mitochondrial integrity, releasing cytochrome c to induce caspase-dependent apoptosis (Meng et al, 2025). Simultaneously, ROS served as a crucial second messenger to activate the NOD-like receptor protein 3 (NLRP3) inflammasome, triggering caspase-1-dependent pyroptosis alongside proinflammatory cytokine release (Li et al, 2025). Lastly, unmitigated ROS drove toxic lipid peroxidation and iron-dependent membrane damage, initiating ferroptosis (Li et al, 2022). Together, ROS-driven apoptosis, pyroptosis, and ferroptosis synergistically depleted functional cardiomyocytes, accelerating HFpEF progression.

#### 2.4.3 Oxidation contributes to hypertrophy

ROS/RNS activated multiple signaling kinases including p38 mitogen-activated protein kinase (p38), extracellular regulated protein kinases 1/2 (ERK1/2), c-Jun N-terminal kinase (JNK), calcium/calmodulin-dependent protein kinase II (CaMKII), and protein kinase C (PKC), triggering pathological myocardial hypertrophy in HFpEF (Emdin et al, 2007; Mohammed et al, 2015; Mukoyama et al, 1991; Nishikimi et al, 2006).

### 2.5 Mitochondrial dysfunction

#### 2.5.1 Reduced ATP production

In HFpEF, impaired mitochondrial oxidative phosphorylation via systemic inflammation caused a severe myocardial adenosine triphosphate (ATP) deficit. Dysfunction of Complexes I and III impaired electron transfer and amplified ROS, while Complex IV inhibition reduced terminal oxygen consumption. Consequently, this electron transport chain failure collapsed the proton gradient, sharply diminishing ATP synthesis, leading to inflammation and worsening myocardial relaxation (Gibb et al, 2025).

#### 2.5.2 Metabolic abnormality

In HFpEF, failing hearts underwent harmful metabolic reprogramming and insulin resistance, displaying distinct profiles marked by altered fatty acid and glucose oxidation. Shifting away from efficient oxidative phosphorylation toward anaerobic glycolysis initially served as a compensatory response to metabolic stress. However, this altered substrate utilization ultimately decreased mitochondrial oxidative capacity, reduced ATP production, and promoted excessive lactate storage, oxidative stress, and toxic lipid accumulation. Consequently, this persistent substrate imbalance disrupted overall bioenergetic efficiency, directly providing a crucial mechanistic link between impaired energy metabolism, progressive mitochondrial damage, and the myocardial energy deficiency characteristic of HFpEF (Gatto et al, 2025).

## 3 Regular treatment of HFpEF

The current clinically recommended treatments for HFpEF mainly include sodium-glucose cotransporter-2 (SGLT2) inhibitors, which was first-line therapy. Mineralocorticoid receptor antagonists (MRAs) and angiotensin receptor-neprilysin inhibitor (ARNI) were second-line drug. Moreover, glucagon-like peptide-1 receptor (GLP-1R) agonists was the treatment for obesity phenotype in HFpEF and MRAs was the treatment for hypertension phenotype in HFpEF. β-adrenergic receptor (β-AR) blockers and calcium channel blockers (CCBs) controlled comorbidities, such as AF in HFpEF.

### 3.1 Sodium-glucose cotransporter-2 inhibitor

In HFpEF, SGLT2 inhibitors exerted cardiovascular protection independently of glycemic control through multiple mechanisms: enhancing osmotic diuresis and natriuresis to relieve volume overload, improving myocardial energetics by shifting fuel utilization toward ketones via activating AMP-activated protein kinase (AMPK), reducing inflammation and oxidative stress via activating sirtuin 1 (SIRT1), suppressing cardiac fibrosis via inhibiting mammalian target of rapamycin complex 1 (mTORC1), and inhibiting the Na^+^-H^+^ exchanger (NHE1) (Jhund, 2022). Clinical trials demonstrated that SGLT2 inhibitors reduced cardiovascular mortality and HF hospitalizations. It increased Kansas City Cardiomyopathy Questionnaire (KCCQ) score and extended 6-min walk distance (6 MWD). It decreased N-terminal B-type brain natriuretic peptide precursor (NT-proBNP) levels and serious adverse event rates, showing favorable safety profiles across diverse phenotype in HFpEF (Santos Guzmán et al, 2025).

### 3.2 Mineralocorticoid receptor antagonist

In HFpEF, MRAs induced renal natriuresis and diuresis, reducing systemic blood pressure without sympathetic reflex activation. It suppressed aldosterone-driven myocardial fibrosis and cardiac stiffness via inhibiting TGF-β signaling and improved cardiac function by attenuating pathological cardiac hypertrophy. It also inhibited mineralocorticoid receptor activation in endothelial cells, reducing oxidative stress via activating Ras-related C3 botulinum toxin substrate 1 (Rac1), and mitigating vascular inflammation (Hiraiwa et al, 2024). Clinical trials found that MRAs significantly reduced HF hospitalization rates. It improved cardiac diastolic parameters and mitigated cardiac structural remodeling and myocardial fibrosis. It enhanced NYHA functional class and exercise tolerance over time. It decreased blood pressure and loop diuretic requirements, suitable for hyptension-phenotype HFpEF, but markedly increased hyperkalemia risk (Capuano et al, 2015).

### 3.3 Angiotensin receptor-neprilysin inhibitor

ARNI treatment attenuated cardiac remodeling and diastolic dysfunction in HFpEF, possibly related to anti-inflammation by down-regulating cell adhesion molecule (intercellular adhesion molecule-1 (ICAM-1) and vascular endothelial cell adhesion molecule-1 (VCAM-1)) expression; Anti-hypertrophy via blocking the phosphorylation of glycogen synthase kinase 3β (GSK-3β); Anti-fibrosis via inhibiting the TGF-β1/Smads pathway (Shi Y. J. et al, 2023). Clinical studies showed ARNI significantly reduced NT-proBNP levels and improved KCCQ score, however it got limited use in reductions in heart failure hospitalizations, cardiovascular mortality, or all-cause mortality (Mattumpuram et al, 2024).

### 3.4 Glucagon-like peptide-1 receptor agonist

GLP-1R agonists mitigate cardiorenal-metabolic disease through complementary cellular mechanisms, including endothelial preservation via activating eNOS, anti-inflammation via inhibiting NLRP3 inflammasome, anti-fibrosis via inhibiting TGF-β/Smad, and decreased adipose and metabolic dysfunction via reducing free fatty acid (FFA). Clinically, it reduced major adverse cardiovascular events, increased KCCQ score and extended 6 MWD. It also induced sustained weight and albuminuria loss and slowed eGFR decline, delivering comprehensive cardiorenal benefits (Hullon et al, 2025).

### 3.5 Other drugs

Both (β-AR) blockers and CCBs reduced heart rate and prolong diastolic filling time, making them useful for controlling ventricular rate in AF. Unlike in HFrEF, β-AR-blockers did not reduce mortality in unselected HFpEF and might worsen chronotropic incompetence. Non-dihydropyridine CCBs were preferred for AF rate control over β-AR-blockers when LVEF was strictly preserved. They were suitable for the comorbidity in HFpEF (Saksena et al, 2025).

### 3.6 Preclinic treatment of HFpEFvia Traditional Chinese Medicine

Due to the “multi-target” effect, the use of TCM for HFpEF has some advantages. Many studies in TCM metabolites and prescriptions have shown that they are effective or potential in combating HFpEF via anti-inflammation, anti-oxidation and improved mitochondrial dysfunction. The evidence can be classified into HFpEF-specific animal evidence (Tables 2, 3) and indirect cardiovascular evidence (Table 4), including computational prediction.

**TABLE 2:** TCM metabolites for HFpEF with different mechanisms.

Ref.	TCM metabolites	Molecular formula	CAS NO.	Model	Route	Dosage	Intervention duration	Groups	Major mechanisms	Molecular mechanisms
Wang et al. (2024)	Astragaloside IV	C_41_H_68_O_14_	84687-43-4	*In vivo* High-fat diet (HFD) and L-NAME-induced HFpEF mice	Oral gavage	40 mg/kg/day	12 weeks	Control; HFpEF; HFpEF + AS-IV	Anti-inflammatory effects and improve mitochondrial dysfunction	CRP ↓IL1RL1↓ GDF15↓ PDH Activity ↑
Dawuti et al. (2023)	Salvianolic acid A	C_26_H_22_O_10_	96574-01-5	*In vivo* HFD and L-NAME-induced HFpEF mice	Oral gavage	2.5, 5, 10 mg/kg/day	4 weeks	Control; HFpEF; HFpEF + SAA	Anti-inflammatory effects	TLR/Myd88/ TRAF/ NF-κB ↓ p38 MAPK/ CREB ↓ IL-6↓ TNF-α↓ COX2↓ iNOS↓
Hao et al (2023)	Periplocin	C_36_H_56_O_13_	13137-64-9	*In vivo* DOCA/ Mononephrectomy-induced HFpEF rats	Oral gavage	1.5 mg/kg/day	8 weeks	Sham/control; DOCA-salt HFpEF; DOCA-salt HFpEF + periplocin	Anti-inflammatory effects	[Ca]_C_^2+^ re-uptake↑ MMP9↓ TGF-β↓ NF-κ B↓ IL-4↑ IFN-γ↓ Treg cells↑
Wei et al (2025)	Limonin	C_26_H_30_O_8_	1180-71-8	*In vivo* HFD and L-NAME-induced HFpEF mice	Dietary	25, 50, 100 mg/kg/day	6 weeks	Control; HFpEF; HFpEF + limonin	Anti-oxidative stress	Fe^2+^↓ P53 ↓ SLC7A11/ GPX4↑ ROS↓ MDA↓ 4-HNE↓ ACSL4↓ COX2↓ ALOX12↓ ALOX15↓
Abudureyimu et al (2023)	Berberine	C_20_H_18_NO_4_	2086-83-1	*In vivo* HFpEF mice induced by HFD and L-NAME	Oral gavage	50 mg/kg	4 weeks	Control; HFpEF; HFpEF + berberine	Improve mitochondrial dysfunction	Drp1↓ p-Drp1 (Ser616) ↓Ca^2+^ homeostasis↑
Mu et al (2025)	Berberine	C_20_H_18_NO_4_	2086-83-1	*In vivo* HFpEF rat model	Oral gavage	100 mg/kg/day	12 weeks	Control; HFpEF; HFpEF + berberine	Anti-oxidative stressand improve mitochondrial dysfunction	AMPK ↑ PGC-1α↑ ROS ↓
Han et al (2024)	Genipin	C_17_H_24_O_10_	24512-63-8	*In vivo*: HFD + L-NAME-induced HFpEF mice	Oral gavage	25,50 mg/kg	8 weeks	Control; HFpEF; HFpEF + geniposide	Anti-oxidative stress	MMP2↓SIRT1↑GSK3β↓ Nrf2↑ HO-1↑ NQO1↑ SOD↑
Yu et al (2026)	Ginsenoside Rb3	C_53_H_90_O_22_	68406-26-8	*In vivo*: HFD + L-NAME-induced HFpEF mice *In vitro*: PE-induced hypertrophic cardiomyocytes	Oral gavage/incubation	Preclinical dose/ Cell dose	Multi-week	Control; HFpEF; HFpEF + G-Rb3; HFpEF + G-Rb3 + SIRT4 inhibitor	Improve mitochondrial dysfunction	SIRT4 ↑ SUCLG1 debutyrylation (K90)↑ TCA cycle flux ↑

**TABLE 3:** TCM prescriptions for HFpEF with different mechanisms.

Ref.	TCM formulations	Model	Route	Dosage	Intervention duration	Groups	Major mechanisms	Molecular mechanisms
Huang et al. (2023)	QiShenYiQi Dropping Pills (Astragali Radix, Salviae Miltiorrhizae Radix ET Rhizoma, Notoginseng Radix Et Rhizoma, and Dalbergiae Odoriferae Lignum)	*In vivo* HFD + L-NAME-induced HFpEF mice	Oral gavage	1.5–3.0 g/kg/day	Multi-week	Control HFpEF HFpEF + QSYQ; HFpEF + QSYQ + AAV-shPKG	Anti-inflammatory effects and improve mitochondrial dysfunction	cGMP-PKG↑ fatty acid metabolism↑
Kang et al. (2024)	Dengzhan sShengmai Capsule (Erigerontis Herba, Ginseng Radix et Rhizoma, Schisandrae Chinensis Fructus, and Ophiopogonis Radix)	*In vivo*: Aldosterone infusion (0.3 μg/h) + uninephrectomy-induced HFpEF mice	Oral gavage	1.5–3.0 g/kg/day	4 weeks	HFpEF HFpEF + DZSMC HFpEF + captopril (positive control)	Anti-inflammatory effects	IL-6 ↓ CRP ↓ IL1RL1 ↓ VCAM ↓ E-selectin ↓ CD31 ↑ eNOS ↑
Shi Y. et al. (2023)	Ling-Gui-Qi-Hua decoction (Poria cocos Wolf Cinnamomum cassia Paeonia veitchii LynchAtractylodes macrocephala Koidz)	*In vivo*: metabolic syndrome-associated HFpEF rats	Oral gavage	1.5–3.0 g/kg/day	8 weeks	Control HFpEF HFpEF + LGQH	Anti-inflammatory effects	TGF-β1↓ Smad3↓ p-Smad2/ Smad3↓ Smad4↓ Smad7↑ ANP↓BNP↓ collagen I ↓
Jiaying et al. (2022)	Shunxin Decoction (Panax ginseng C.A.Mey.; Astragalus mongholicus Bunge; Atractylodes macrocephala Koidz.; Poria; Chuanxiong Rhizoma; Paeoniae Radix Rubra; Lepidii Semen; Salvia miltiorrhiza Bunge; Glycyrrhizae Radix et Rhizoma)	*In vivo*: abdominal aorta constriction (AAC)-induced HFpEF rats	Oral gavage	High-dose and low-dose (Shunxin decoction)	14 days	Control Sham HFpEF HFpEF + Shunxin high-dose; HFpEF + Shunxin HFpEF + low-dose; QLQX capsule (positive control)	Anti-inflammatory effects	NPRA↑ GC↑ eNOS↑ cGMP ↑ PKG I↑ PDE5A ↓
Yuan et al. (2023)	Qidan Formula (Astragalus mongholicusBunge, Codonopsis pilosula (Franch.) Nannf., Polygonatum kingianum Collett and Hemsl./Polygonatum sibiricum F. Delaroche, Salvia miltiorrhiza Bunge, Paeonia lactiflora Pall./Paeonia veitchii (Lynch) D.Y. Hong and K.Y. Pan, and Curcuma aromatica Salisb./Curcuma kwangsiensis S.G. Lee and C.F. Liang)	*In vivo* SHR rats fed with high-fat/high-sucrose diet + STZ (25 mg/kg, i.p.)-induced hypertensive and diabetic HFpEF rat	Oral gavage	Preclinical dose (high/medium/Low dose groups)	14 weeks (or multi-week treatment post-induction)	Control (WKY/Control); HFpEF Qidan formula	Anti-inflammatory effects	Ang II ↓ AT1R↓ TGF-β1↓ P-Smad2↓ P-Smad3↓ MMP-9 ↓
Liang et al. (2026)	Mahuang Decoction (Ephedra sinica Stapf, Cinnamomum cassia Presl, Prunus armeniaca L., and Glycyrrhiza uralensis FischexDC.)	*In vivo*: HFD + L-NAME-induced HFpEF mice *In vitro*: AC16 cardiomyocytes	Oral gavage	Preclinical standard dosage	2 weeks (following 10 weeks of HFD + L-NAME induction)	Control (normal); HFpEF HFpEF + Mahuang decoction	Anti-inflammatory effects	p38 MAPK pathway ↓ AQP4 ↓ AQP2 ↓ AQP5 ↑ PCSK6 ↑ Corin ↑ p-NPR-A↑
Hao et al. (2025)	Qiliqiangxin Formula (Astragalus mongholicus Bunge,Panax ginseng C.A.Mey., Aconitum carmichaelii Debeaux, salvia miltiorrhiza Bunge ,Descurainia sophia (L.) Webb ex Prantl and/or Lepidium apetalum Willd, Alisma rhizome,Polygonatum odoratum (Mill.) Druce, Cinnamomum cassia (L.) J.Presl, Carthamus tinctorius L., Periploca sepium Bunge, and Citri Reticulatae Pericarpium)	*In vivo*: unilateral nephrectomy + DOCA pellet + high-salt diet-induced HFpEF rat	Oral gavage	Preclinical dosage	Multi-week treatment period post-induction	Control (Sham) HFpEF HFpEF + QLQX	Anti-inflammatory effects	eNOS ↑NO ↑ cGMP-PKG ↑ PKG ↑

**TABLE 4:** TCM metabolites for indirect models with potential mechanisms.

Ref.	TCM metabolites	Molecular formula	CAS NO.	Model	Route	Dosage	Intervention duration	Groups	Major mechanisms	Molecular mechanisms
Jiang et al. (2019)	Astragaloside IV	C_41_H_68_O_14_	84687-43-4	*In vivo* Ischemia/reperfusion (I/R)-induced HF rats	Oral gavage	40 mg/kg/day	1 week	Sham; I/R-HF; I/R-HF + AS-IV	Anti-oxidative stress effects	Keap1 ↓ CAT↑ Nrf2↑ HO-1↑ SOD↑ SDH↑
Nie et al. (2019)	Astragaloside IV	C_41_H_68_O_14_	84687-43-4	*In vivo* Pressure overload-induced cardiac hypertrophy (CH) rats	Oral gavage	40 mg/kg/day	8 weeks	Sham CH CH + AS-IV	Anti-oxidative stress effects	Nrf2 ↑ HO-1 ↑
Zang et al. (2020)	Astragaloside IV	C_41_H_68_O_14_	84687-43-4	*In vivo*: Isoprenaline (ISO)-induced myocardial fibrosis (MF) rats *In vitro*: Cardiac fibroblasts treated with ISO/ROS	Injection	80 mg/kg/day	4 weeks	Control MF MF + AS-IV	Anti-inflammatory effects and anti-oxidative stress effects	ROS ↓ CT-1 ↓ collagen I ↓ Collagen III ↓
Jin et al. (2026)	Astragaloside IV	C_41_H_68_O_14_	84687-43-4	*In vivo*: ISO-induced heart failure rats *In vitro*: ISO-treated cardiomyocytes	Intragastrically	40, 80 mg/kg/day	1 week	Control; HF HF + AS-IV	Anti-inflammatory effects	p38 MAPK↓ NF-κB↓ TNF-α↓ IL-6↓ IL-1β↓
Dai et al (2017)	Astragaloside IV	C_41_H_68_O_14_	84687-43-4	*In vivo* Pressure overload/transverse aortic constriction (TAC)-induced cardiac remodeling mice *In vitro*: H_2_O_2_/ROS-induced cardiomyocytes	AS-IV: intragastrically ATO: Intraperitoneally	AS-IV: 40, 80 mg/kg/day ATO: 5 mg/kg	1 week	Sham; TAC TAC + AS-IV	Anti-inflammatory effects and anti-oxidative stress effects	ROS↓ p38 MAPK↓ERK1/2↓ JNK↓
Wang et al. (2019)	Ginsenoside Re	C_48_H_82_O_18_	52286-59-6	*In vivo*: ISO-induced myocardial fibrosis and HF rats	Oral gavage	50 mg/kg/day	4 weeks	Control HF HF + ginsenoside Re	Anti-inflammatory effects	TGF-β 1↓ p-Smad3↓ CollagenI↓
Zhang et al. (2016)	Ginsenoside Rg3/ginsenoside Rh1	C_42_H_72_O_13_/ C_36_H_62_O_9_	14197-60-5/ 63223-86-9	*In vivo* Rat myocardial I/R injury mice (Rg3) and ISO-induced cardiac injury (CI)mice (Rh1)	Intragastrically	Low-dose:5 mg/kg/day High-dose:20 mg/kg/day	1week	Control/ I/R CI I/R + Rg3 CI + Rh1	Anti-inflammatory effects Anti-oxidative stress effects	p53↓ Bcl-2↑ Bax↓ Cleaved caspase-3↓ TNF-α↓IL-1β ↓
Ge et al (2025)	Baicalin	C_21_H_18_O_11_	21967-41-9	*In vivo*: ISO-induced HF mice	Intraperitoneally	100 mg/kg/day	4weeks	Control; HF HF + baicalin	Anti-oxidative stress	8-OHdG ↓3-NT↓ NADPH ↓ NOX2↓
Guo et al (2025)	Quercetin	C_15_H_10_O_7_	117-39-5	*In vitro*/*In vivo*: HF-related experimental model	Intraperitoneally	15 mg/kg/day	4weeks	Control; HF HF+ Quercetin; HF+ Isoquercitrin comparison	Anti-inflammatory effects Anti-oxidative stress effects	MAPK ↓ERK ↓ p38 ↓NF-κB↓Bcl-2↑ Bax↓ Caspase-3↓
Chen X. et al. (2022)	Paeonol	C_9_H_10_O_3_	552-41-0	*In vivo* TAC-induced pressure overload HF mice	Intraperitoneally	10, 20, 50 mg/kg/day	4weeks	Sham TAC TAC + paeonol	Anti-inflammatory effects	ERK1/2 ↓ NK ↓
Ding et al. (2023)	Paeonol	C_9_H_10_O_3_	552-41-0	*In vivo*/*In vitro*: Doxorubicin-Induced cardiac injury model	Oral gavage	75, 150, 300 mg/kg/day	2weeks	Control; doxorubicin doxorubicin + paeonol	Improve mitochondrial dysfunction	Mfn2 ↑
Chen L. et al. (2024)	Tanshinone IIA	C_19_H_18_O_3_	568-72-9	In silico Network pharmacology prediction for HFpEF	NR	NR	NR	NR	Anti-inflammatory effects	CRP↓IL6↓ TNF-α↓ MDA↓ SOD↑ GSH-Px↑

### 3.7 HFpEF-specific animal evidence

#### 3.7.1 TCM metabolites

Astragaloside IV (AS-IV) is a characteristic triterpenoid saponin isolated from the dried root of *Astragalus membranaceus* (Fisch.) Bunge [Fabaceae; Astragali radix]. It serves as the principal bioactive marker metabolite of Astragali radix for the treatment of HFpEF. AS-IV alleviated HFpEF by suppressing inflammation and correcting myocardial metabolic dysfunction via improving mitochondrial Complex I activity, enhancing ATP production, and restoring glucose metabolism (Wang et al, 2024). Preclinical studies showed AS-IV was 2.2%–7.4% oral bioavailability (Gu et al, 2004) and reduced omeprazole exposure via inducing intestinal P-glycoprotein (*P-gp*) to decrease absorption and hepatic cytochrome P450 3A4 (CYP3A4) to accelerate metabolism (Liu et al, 2019). Clinical studies showed AS-IV exhibited linear, non-accumulating pharmacokinetics with a short half-life, and was well-tolerated with mild, transient adverse events in short-term (Xu et al, 2013). Therefore, further studies are needed to establish its long-term safety and interactions with standard GDMT profile for its clinical translation.

Salvianolic acid A (SAA) is a water-soluble phenolic acid metabolite extracted from the dried root and rhizome of *Salvia miltiorrhiza* Bunge [Lamiaceae; Salviae miltiorrhizae radix et rhizoma]. SAA effectively improved cardiac function and comorbidities in HFpEF, independent of blood pressure regulation. Echocardiography revealed that SAA inhibited pathological myocardial hypertrophy and enhanced left ventricular (LV) diastolic function by reducing myocardial wall thickness and mass. SAA suppressed inflammation by downregulating IL-6, TNF-α, cyclooxygenase-2 (COX2), and iNOS. It inhibited the Toll-like receptor (TLR)/myeloid differentiation factor 88 (Myd88)/TNF receptor-associated factor (TRAF)/nuclear factor κB (NF-κB) signaling pathway and p38/cAMP response element-binding protein (CREB) activation (Dawuti et al, 2023). Preclinical studies reported SAA was 0.39%–0.52% oral bioavailability (Sun et al, 2018). The Clinical studies validated physiologically based pharmacokinetic (PBPK) modeling accurately predicted well-tolerated SAA’s organic anion transporting polypeptide 1B1 (OATP1B1) and *P-gp* saturation. Besides, it demonstrated good safety and tolerability with no clinically meaningful hepatic and renal changes (Chen J. et al, 2022). Further studies are needed to establish its long-term safety and interactions with standard GDMT profile for its clinical translation.

Periplocin is a cardiac glycoside metabolite derived from the dried root bark of *Periploca sepium* Bunge [Apocynaceae; Periplocae cortex]. Periplocin significantly improved cardiac structure and function in HFpEF. It protected against hypertensive cardiac remodeling and dysfunction by reducing heart size, cardiomyocyte cross-sectional area, heart weight (HW)/body weight (BW) ratio, and NT-proBNP levels. Periplocin reduced matrix metallopeptidase 9 (MMP9), TGF-β, and NF-κB levels. It also exerted cardio-prescending effects via enhancing calcium ion reuptake to accelerate contraction and relaxation. Additionally, periplocin suppressed inflammation through increasing interleukin-4 (IL-4) and Treg cells while reducing IFN-γ (Hao et al, 2023). Preclinical studies showed periplocin exhibited low oral bioavailability driven by rapid deglycosylation, *P-gp* efflux, a narrow therapeutic window, and dose-dependent nonlinear pharmacokinetic clearance (Zhao et al, 2026). Preclinical and clinical studies showed *P-gp* and organic anion transporting polypeptides (OATPs) were implicated in periplocin’s absorption and biliary excretion, raising potential botanical drug-drug interaction concerns (Liang et al, 2014). Further studies are needed to define its therapeutic window, long-term safety, and compatibility with GDMT for its clinical translation.

Limonin is a limonoid metabolite extracted from the dried immature fruit of *Citrus aurantium* L. [Rutaceae; Aurantii fructus immaturus]. Limonin exhibited anti-inflammatory and antioxidant effects to treat HFpEF. It improved dyslipidemia, hypertension, and slowed weight gain in HFpEF. Limonin alleviated concentric myocardial hypertrophy by reversing cell disarray and reducing collagen deposition via inflammation. It combated ferroptosis via enhancing the solute carrier family 7 member 11 (SLC7A11)/glutathione peroxidase 4 (GPX4) axis, regulating iron homeostasis (inhibiting transferrin receptor (TFRC), increasing ferritin heavy chain 1 (FTH)), and activating the nuclear factor erythroid 2-related factor 2(Nrf2)/heme oxygenase-1(HO-1) pathway. Furthermore, limonin reduced cardiac fat accumulation and inhibited lipid peroxidation by reversing the overexpression of acyl-CoA synthetase long chain family member 4 (ACSL4), COX2, arachidonate 12-lipoxygenase (ALOX12) and arachidonate 15-lipoxygenase (ALOX15) enzymes (Wei et al, 2025). Preclinical studies have raised concerns regarding poor oral bioavailability due to low water solubility, rapid elimination half-life, extensive hepatic metabolism, and complex cytochrome (CYP) enzyme interactions, emphasizing that scarce clinical data currently limited its direct clinical drug translation (Fan et al, 2019). Further studies are required to define its compatibility with GDMT.

Ginsenosides are dammarane-type triterpenoid saponin metabolites extracted from the dried root and rhizome of *Panax ginseng* C.A.Mey. [Araliaceae; Ginseng radix et rhizoma]. Ginseng exerted beneficial effects on heart disease through its antioxidant activity, reduced platelet aggregation, and regulation of vascular function and ion channels (Lee and Kim, 2014). Its primary active metabolites, ginsenosides, play distinct roles in cardioprotection. Ginsenoside Rb3 improved HFpEF by targeting sirtuin 4 (SIRT4) to reverse succinate-CoA ligase, GDP-forming, alpha subunit (SUCLG1) hyperbutyrylation, restoring tricarboxylic Acid (TCA) cycle flux and ATP production (Yu et al, 2026). Clinical studies showed ginsenosides underwent extensive gut microbiota and hepatic metabolism, generating compound K that enhanced insulin sensitivity and suppressed lipid accumulation (Hu et al, 2013). Ginsenosides exhibited linear pharmacokinetics, while compound K exhibited delayed peak exposure. It also showed low risk for CYP-mediated drug interactions, while P*-gp* efflux restricted native saponin absorption, favoring metabolite Compound K (Choi et al, 2020). Further studies are needed to clarify the roles of specific ginsenosides in systemic exposure, long-term safety, and compatibility with GDMT for their clinical translation.

Berberine is an isoquinoline alkaloid metabolite extracted from the dried rhizome of *Coptis chinensis* Franch. [Ranunculaceae; Coptidis rhizoma]. Berberine has demonstrated significance in treating HFpEF (Wang et al, 2017). In HFpEF, berberine was shown to improve diastolic function, with early-to-atrial filling velocity (E/A) ratio, early filling velocity-to-early diastolic mitral annular velocity (E/e′) ratio, and LV mass significantly improved. This was achieved by suppressing dynamin-related protein 1 (Drp1) Ser616 phosphorylation and mitochondrial translocation, which reduced mitochondrial fragmentation, restored ultrastructure and inhibited abnormal mitochondrial fission (Abudureyimu et al, 2023). In HFpEF, berberine increased phosphated AMPK (p-AMPK) and peroxisome proliferator-activated receptor gamma coactivator 1-alpha (PGC-1α) expression, reduced mitochondrial ROS production, and improved transcription factor A, mitochondrial (TFAM)/nuclear respiratory factor 1 (NRF1)-mediated mitochondrial biosynthesis (Mu et al, 2025). Additionally, berberine restored the expression of autophagy-related proteins (LC3B, Atg5, Atg7) and reduced p62 accumulation, thereby clearing damaged mitochondria and toxic protein aggregates in HFpEF (Abudureyimu et al., 2023; Zhu et al, 2020). Preclinical and clinical studies showed berberine was poor oral bioavailability (<1%) due to low solubility/permeability, P*-gp* efflux, and extensive metabolism. However, modified formulations could markedly enhance absorption. Its short-term use was generally well tolerated, though gastrointestinal adverse effects occur (Khoshandam et al., 2022). Importantly, Clinical studies reported that repeated administration inhibited CYP2D6, CYP2C9, and CYP3A4, indicating a potential botanical drug-drug interaction (Guo et al, 2012). Further studies are needed to establish relevant exposure, long-term safety, and compatibility with GDMT for their clinical translation.

Genipin is an iridoid aglycone metabolite extracted from the dried ripe fruit of *Gardeniajasminoides* Ellis [Rubiaceae; Gardeniae fructus]. Genipin exhibited diverse pharmacological activities, including hepatoprotective, anti-diabetic, and anti-myocardial dysfunction effects. In the cardiovascular field, it showed suitable for treating HFpEF by regulating oxidative stress (Liu et al, 2022; Mu et al., 2025). Genipin improved the E/A ratio, enhanced exercise tolerance, and reduced myocardial fibrosis in HFpEF. Molecular docking and SPR techniques confirmed that genipin directly bound to matrix metallopeptidase 2 (MMP2). This interaction upregulated SIRT1 expression and inhibited GSK3β activity, resulting in nuclear translocation of Nrf2 to initiate the transcription of antioxidant enzymes (HO-1, NAD(P)H quinone dehydrogenase 1 (NQO1), and superoxide dismutase (SOD)), ultimately alleviating oxidative stress (Han et al, 2024). Preclinical studies showed orally administered genipin underwent extensive metabolism to genipin sulfate, with no parent metabolite detected in circulating plasma. Dose-dependent toxicity has been reported, with high oral doses causing substantial mortality, underscoring the need to define its therapeutic window (Hou et al, 2008). Further clinical studies are required to establish its pharmacokinetics, therapeutic window, long-term safety, botanical drug-drug interaction potential and compatibility with GDMT for their clinical translation.

#### 3.7.2 TCM prescriptions

Qi-Shen-Yi-Qi (QSQY) Dropping Pills significantly improved cardiac function in HFpEF, LV compliance and hemodynamics. Its mechanisms primarily involved activation of the NO-cGMP-PKG pathway, inhibition of microvascular endothelial inflammation, suppression of endothelial-mesenchymal transition, and attenuation of oxidative stress (Huang et al, 2021). They integrated cardiac multi-omics (transcriptomics, proteomics, metabolomics) in HFpEF to predict QSYQ’s mechanisms. PKG knockdown abolished most cardioprotective effects, though certain hypertrophy indices reduced, confirming PKG as a core mediator while suggesting ancillary pathways (Huang et al, 2023). Preclinical studies found QSQY exhibited complex multi-metabolite pharmacokinetics with rapid absorption, wide tissue distribution, extensive metabolism, and varied elimination rates (Shao et al, 2017). Clinical studies exhibited favorable overall safety with low incidence of adverse effects and no severe treatment-related events (Xingmeng et al, 2023). Further studies are needed to define its long-term safety and compatibility with GDMT for its clinical translation.

Deng-Zhan-Sheng-Mai (DZSM) Capsules is mainly used for the treatment of cardiovascular and cerebrovascular diseases (Xu et al, 2021). Network pharmacology prediction combined with *in vitro* experiments, demonstrated that DZSM not only significantly improved cardiac function in HFpEF, as evidenced by reduced myocardial hypertrophy, reversal of E/A peak inversion, and decreased a′/e′ and E/e′ ratios, but also ameliorated myocardial fiber disarray and mitochondrial morphology. DZSM inhibited chronic systemic inflammation (reducing serum levels of IL1RL1, C-reactive protein (CRP), and IL-6) and microvascular inflammation (reducing VCAM-1 and E-selectin, while increasing CD31 and eNOS expression) (Kang et al, 2024). Preclinical studies found DZSM exhibited multi-metabolite pharmacokinetic characteristics, showing rapid oral absorption and extensive metabolic transformation (Ma et al, 2023). DZSM altered clopidogrel pharmacokinetics by inhibiting P-*gp* efflux transport and CYP450-mediated metabolism, reducing active metabolite plasma exposure (Chen et al, 2016). Clinical studies demonstrated good safety with low adverse events in HF, though long-term safety requires further evaluation (Lu et al, 2025). Further studies are needed to define its long-term safety and compatibility with GDMT for its clinical translation.

Ling-Gui-Qi-Hua (LGQH) is a formula derived from Ling-Gui-Zhu-Gan decoction. A previous study demonstrated that LGQH effectively alleviated LV remodeling and diastolic dysfunction in HFpEF. Meanwhile, LGQH exerted anti-myocardial fibrosis effects by blocking the TGF-β1/Smads signaling pathway. Furthermore, molecular docking between the active metabolites of LGQH and key proteins of the TGF-β1/Smads pathway (including TGF-β1, Smad2/3/4/7, α-smooth muscle actin (α-SMA), and Collagen I/III) provided support for subsequent investigations into the detailed mechanism (Shi Y. et al, 2023). The available research was extremely limited regarding LGQH. Only 13 active metabolites have been identified in pharmacokinetic studies, and all investigations were confined to animal pharmacodynamics (Shi Y. et al, 2023), with an absence of reports on pharmacokinetics, safety, and compatibility with GDMT.

Shunxin decoction has been used in Chinese patients of HFpEF for several years (Dou et al, 2019). Through network pharmacology prediction combined with experimental validation, on the one hand, it was demonstrated that Shunxin decoction activated the cGMP-PKG signaling pathway (up-regulating natriuretic peptide receptor A (NPRA), guanylate cyclase (GC), and eNOS) to increase cGMP levels. On the other hand, downregulation of PDE5A by Shunxin decoction inhibited cGMP degradation, leading to activation of PKG. As a result, it reversed pathological myocardial hypertrophy, enhanced myocardial compliance, effectively improved LV diastolic function, and alleviated ultrastructural damage in HFpEF (Jiaying et al, 2022). Although the Shunxin decoction was the original clinical form, only one study addressed its sustained-release granules rather than the decoction itself, demonstrating sustained release and enhanced bioavailability (Dou et al, 2019). Nevertheless, the safety of Shunxin decoction remained unclear, and no compatibility with GDMT reported.

Qidan formula has good clinical effects in the treatment of cardiovascular diseases. The Qidan formula exerted significant therapeutic effects on hypertension and diabetes phenotype-HFpEF by inhibiting the angiotensin II (Ang II)/TGF-β1/Smad2/3 signaling pathway, thereby alleviating myocardial fibrosis and improving cardiac diastolic function and myocardial pathological morphology (Yuan et al, 2023). The pharmacokinetics, safety, and compatibility with GDMT remained unverified.

Mahuang decoction is mainly used to induce sweating, release the exterior, and ventilate the lungs to relieve asthma. However, Mahuang decoction ameliorated cardiac structure and function in HFpEF via multi-organ coordinated regulation of fluid metabolism. It inhibited the p38 mitogen-activated protein kinase (MAPK) signaling pathway, downregulated cardiac aquaporin-4 (AQP4) and renal aquaporin-2 (AQP2) expression to alleviate tissue edema, and upregulated cutaneous aquaporin-5 (AQP5) and corinto to promote sweat excretion. In addition, Mahuang decoction activated the proprotein convertase subtilisin/kexin type 6 (PCSK6)/Corin/atrial natriuretic peptide (ANP) pathway, restoring ANP bioactivity and enhancing natriuretic and diuretic effects (Liang et al, 2026). This study provided the first experimental evidence that Mahuang decoction coordinately regulated fluid balance via the heart-kidney-skin axis, offering a novel therapeutic strategy for HFpEF. It showed multi-metabolite pharmacokinetics with rapid oral absorption and differential elimination rates (Huang et al, 2020). The safety and compatibility with GDMT remained unveiled.

Qiliqiangxin (QLQX) formula exerted cardioprotective effects through multi-pathway and multi-target mechanisms. Through network pharmacology and multi-omics comparative analysis, the cGMP-PKG signaling pathway was identified as a key target of QLQX in the treatment of HFpEF. Subsequent *in vivo* experiments further validated that QLQX activated the cGMP-PKG signaling pathway, thereby improving diastolic function, myocardial remodeling, and endothelial function (Hao et al, 2025). QLQX formula exhibited pharmacokinetics of multiple metabolites, characterized by rapid absorption, wide distribution and rapid clearance of many active metabolites, while toxic alkaloids maintained low systemic exposure. Clinically, it demonstrated a favorable safety profile with low adverse event rates and tolerability to standard HF therapies (Zhang et al, 2019).

### 3.8 Indirect cardiovascular evidence

Astragaloside IV exerts potential therapeutic benefits in HFpEF via anti-inflammation and anti-oxidation mechanisms. It also activated the kelch-like ECH-associated protein 1 (Keap1)/Nrf2 pathway, promoting antioxidant gene transcription (HO-1, sSOD, and catalase (CAT)) to alleviate ischemia-reperfusion (I/R) injury (Jiang et al, 2019). AS-IV suppressed hypertrophy by upregulating Nrf2/HO-1 (Nie et al, 2019). AS-IV also protected against fibrosis by reversing ROS-induced MAPK (Dai et al, 2017) and cardiotrophin-1 (CT-1) activation (Zang et al, 2020). AS-IV protected against arsenic trioxide (ATO)-induced cardiac toxicity by inhibiting p38MAPK/NF-κB pathway (Jin et al, 2026).

Ginsenoside exerts potential therapeutic benefits in HFpEF via anti-inflammation and antioxidant mechanisms. Ginsenoside Rg3 improved cardiac functionwith myocardial I/R injury, while ginsenoside Rh1 alleviated isoprenaline-induced myocardial damage (Zhang et al, 2016). Ginsenoside Re inhibited isoproterenol (ISO)-induced myocardial fibrosis and HF by reducing TGF-β1 levels and suppressing Smad3 phosphorylation, thereby decreasing collagen I expression (Wang et al, 2019).

Genipin exerts potential therapeutic benefits in HFpEF via anti-inflammation mechanisms. Genipin exerted anti-apoptotic and anti-inflammatory effects by inhibiting the COX2-PGD2 and MDM2-p53 pathways, preventing post-myocardial infarction structural remodeling (Zhao et al, 2022).

Baicalin is a flavonoid glycoside metabolite extracted from the dried root of *Scutellaria baicalensis* Georgi [Lamiaceae; Scutellariae radix]. Baicalin exerts potential therapeutic benefits in HFpEF via antioxidant properties. Baicalin demonstrated anti-hypertrophic and anti-fibrotic effects. It improved LV function and wall thickness but decreased brain natriuretic peptide (BNP) and ANP levels via binding to NADPH oxidase 2 (NOX2) robustly, which prevented oxidative stress by significantly lowering myocardial 8-OHdG and 3-NT levels (Ge et al, 2025).

Quercetin is a flavonol metabolite extracted from the dried root of *A. membranaceus* (Fisch.) Bunge [Fabaceae; Astragali radix]. Quercetin exhibits potential in treating HFpEF via antioxidant and anti-inflammatory effects. Quercetin counteracted myocardial fibrosis by inhibiting the MAPK inflammatory pathway and alleviating oxidative stress (Long et al, 2025). In ISO-induced cell injury models, it significantly reduced the Bax/Bcl-2 ratio and caspase-3 expression by inhibiting extracellular signal-regulated kinase (ERK) and p38 phosphorylation (Ma et al, 2025). Quercetin improved cardiomyocyte survival and microvascular function by activating the protein kinase B (Akt)/B-cell lymphoma 2 (Bcl-2) signaling pathway, which reduced apoptosis and inhibited ventricular remodeling in HF (Hu et al., 2025). It also prevented the progression of hypertension, myocardial hypertrophy, and HF via inhibition of MAPK and NF-κB pathways (Guo et al, 2025).

Paeonol is a phenol metabolite extracted from the dried root bark of *Paeonia suffruticosa* Andrews [Paeoniaceae; Moutan cortex]. Paeonol gets potential in treating HFpEF via improving mitochondrial dysfunction and inhibiting inflammation. Paeonol significantly improved cardiac function, suppressed the expression of myocardial hypertrophy markers ANP and BNP, and alleviated myocardial cell apoptosis and fibrosis by inhibiting the ERK1/2/c-Jun N-terminal kinase (JNK) signaling pathway (Chen X. et al, 2022). It significantly improved the cardiac function and mitochondrial function via restoring expression of mitochondrial fusion protein 2 (Mfn2) in doxorubicin-treated cardiomyocytes, both *in vivo* and *in vitro* (Ding et al, 2023).

Tanshinone IIA is a lipophilic bioactive diterpenoid metabolite extracted from the dried root and rhizome of *Salvia miltiorrhiza* Bunge [Lamiaceae; Salviae miltiorrhizae radix et rhizoma] (Gao et al, 2012). Unlike conventional anti-heart failure drugs, tanshinone IIA significantly enhanced autophagy while inhibiting apoptosis via the AMPK/mammalian target of rapamycin (mTOR) pathway, helping to clear toxic protein aggregated that exacerbated diastolic dysfunction (Chen et al, 2015). Based on the available evidence, tanshinone IIA demonstrates promising therapeutic potential for HFpEF, though direct studies are limited. Network pharmacology predicted tanshinone IIA as a core active metabolite of the Astragalus-Salvia botanical drug pair against HFpEF, potentially via anti-inflammatory and anti-apoptotic pathways (Chen L., et al, 2024).

The pharmacokinetics, safety profiles, and interactions with standard GDMT of all the indirect cardiovascular evidence need further studies after they will be really and effective in HFpEF.

## 4 Clinical studies on traditional Chinese medicine for the treatment of HFpEF

As TCM gains increasing attention in cardiovascular therapy, a growing number of studies have investigated its role in HFpEF (Table 5) in recent years. Guided by TCM principles such as “invigorating qi”, “activating blood circulation,” “warming yang,” and “resolving phlegm and blood stasis,” various botanical prescriptions have demonstrated TCM could enhance the therapeutic efficacy of GDMT as adjuvant treatment, which were effective in improving cardiac function and attenuating myocardial remodeling via modulating inflammation and oxidation, including decreasing NT-proBNP levels and inflammatory factors as well as increasing the anti-oxidative enzymes.

**TABLE 5:** Clinical studies on TCM for HFpEF.

Ref.	TCM (Composition)	Product standard	Study Design (registered)	Patients	Sample size calculation	Intervention (Dose)	Endpoints	Adverse event	Limitations
Liu et al. (2023)	Shexiang Baoxin Pill (SXBX) (Moschus, Ginseng Radix et Rhizoma, Styrax, Cinnamomi Cortex, Bufonis Venenum, Bovis Calculus Artifactus, and Borneolum Syntheticum)	Shanghai Hutchison Pharmaceutical Company National Medicine batch No. 210304	Single-center, double-blind, RCT China clinical trials registry center registration number: ChiCTR2100054322	HFpEF NHYA heart function: class II–III LVEF ≥ 50% Age 40–80 years	Yes N = 63 (21 per group) 55 completed	Conventional HF drugs + home-based exercise training + Shexiang Baoxin Pill treatment (two pills three timesa day) v.s. conventional HF drugs + homebased exercise training treatment v.s. conventional HF drugs oral 12 weeks	↑ peak VO_2_ ↑ AT ↑ 6 MWT ↓PSQI ↑ QoL	No report	Small sample size Single center Short-termeffects Sepcial background without mid-term efficacy
Zhang and Wang (2024)	Tinglu Yixin Prescription (Tinglizi, Luxiancao, Curcuma zedoaria, Poria cocos, raw Astragalus membranaceus, Salvia miltiorrhiza, Rhodiola, Lulutong, Plantain botanical, Poria, Fried Atractylodes, Yuzhu, Cinnamon Twig, Broussonetia papyrifera, Ziziphus jujuba)	Pharmacopoeia of PRC in 2020	Randomized,Parallel-group study China clinical trials registry center registration number: ChiCTR2200058512	HFpEF patients with type 2 diabetes LVEF ≥ 50% BNP ≥ 35 pg/mL or NT-proBNP ≥ 125 pg/mL NHYA heart function: class II–III Age 18–80 years	Yes N = 108 (54 per group) 104 completed	Basic treatment for HF and diabetes + Tinglu Yixin prescription (100 mL twice daily) v.s. basic treatment for HF and diabetes oral 12 weeks	↑ cardiac response (90.4% vs. 67.3%) ↓ E/e′ ↓ NT-proBNP ↓ HbA1c ↑ 6 MWD ↑ QoL	No serious adverse events	Small sample size Single center Short-termeffects Lack blinded design Not included primary endpoint
Yang et al (2025)	San-jia-fu-mai Decoction (SJFM) (Glycyrrhizae radix et rhizoma praeparata cummelle (Zhi-GanCao), rehmanniae radix (Sheng-Di-Huang), paeoniae radix alba(Sheng-Bai-Shao), ophiopogonis radix (Mai-Dong), asini corii colla (E-Jiao), cannabis fructus (Ma-Ren), ostreae concha (ShengMu-Li), trionycis carapax (Sheng-Bie-Jia),testudinis carapax etplastrum(Sheng-Gui-Ban))	Pharmacopoeia of PRC in 2020	Double-blind Placebo-controlled RCT Registered at http://itmctr.ccebtcm.org.cn/	HFpEF with atrial fibrillation LVEF ≥ 50% NT-proBNP≥ 365 pg/mL E/e′ ≥ 15 Age 46–80 years	Yes N = 110 (55 per group)	SJFM (100 mL twice daily) + standard therapy (10 mg once daily) v.s. placebo + standard therapy oral 12 weeks	↑ KCCQ grade (94.12% vs. 68.63%) ↑ KCCQ score ↓ E/e′ ↓ NT-proBNP ↓ sRAGE	No serious adverse events	Small sample size Single center Short-termeffects Simple endpoints No reports of follow-up loss
Chen et al. (2024)	Jianxin Granule (Mao Dongqing, Notoginseng, Safflower, Salvia miltiorrhiza, Borneol, Aquilaria, and Lycopodium)	Min Yao Zhi Zi Z20100010	Open-label, single-center, parallel-group clinical trial Registered no mention	HFpEF LVEF ≥ 50% Age 35–75 years	Yes N = 98 (49 per group) 94 completed	Jianxin Granule (10 g three times daily) + standard therapy v.s. standard therapy oral 8 weeks	↓ CRP ↓ TNF-α ↓ IL-6 ↓ MDA ↑ GSH-Px SOD n.s ↓ NT-proBNP ↓ TCM syndromes ↑ cardiac function	No serious adverse events	Small sample size Single center Short-termeffects Simple endpoints Lack of a placebo-controlled or randomized, blinded design Incomplete long-term safety data
Li et al. (2026)	QSYQ Dripping Pills (Astragali Radix, Salviae Miltiorrhizae Radix ET Rhizoma, Notoginseng Radix Et Rhizoma, and Dalbergiae Odoriferae Lignum)	Tasly Pharma, Tianjin, China; Z20010130	Open-label, evaluator-blinded RCT Registered no mention	HFpEF LVEF ≥ 50%, BNP ≥ 35 pg/mL or NT-proBNP ≥ 125 pg/mL,baseline 6 MWD between 150-450 m Age ≥ 65 years	No N = 84 (42 per group)	QSYQ dropping pills (0.5 g/trip, 3 times daily) + basic HFpEF therapy v.s. placebo + basic HFpEF therapy oral 12 weeks	↑ 6 MWD ↑ SPPB ↑ KCCQ ↓ LAVI NT-proBNP n.s	No serious adverse events	Small sample size Single center no placebo Short-term effects Lack of key biomarker evidence Incomplete long-term safety data No reports of follow-up loss
Yu et al (2026)	Shenzhu Xinkang Decoction (Codonopsis pilosula, Ophiopogon japonicus, fried Astragalus membranaceus, Lepidium sativum seeds, Salvia miltiorrhiza, of Panax notoginseng, of Curcuma longa, Lycopi Herba, Polygonati Rhizoma, Schisandra, Polygonatum odoratum)	product by Affiliated Hospital of Hunan Academy of Chinese Medicine	Single-center, randomized, single-blind, parallel-group study Registered no mention	HFpEF LVEF ≥ 50% BNP ≥ 35 pg/mL or NT-proBNP ≥ 125 pg/mL NHYA heart function: class II–III ACC/AHA classC Age 50–75 years	Yes N = 105 (≥52per group) 105 completed	Shenzhu Xinkang decoction (200 mL twice daily) + dapagliflozin (once daily) + sacubitril/valsartan (twice daily) v.s. dapagliflozin (once daily) + sacubitril/ valsartan (twicedaily) oral 16 weeks	↑ diastolic function ↓ LAVI ↓ epicardial fat thickness ↓ NT-proBNP ↓ inflammatory markers	No serious adverse events	Small sample size Single center Short-termeffects Absence of efficacy endpoints reflecting myocardial fibrosis Exist outcome bias

HF, heart failure; peak VO_2_, peak oxygen uptake; AT, anaerobic threshold; 6 MWT, 6-min walk test; PSQI, Pittsburgh Sleep Quality Index (reflects sleep quality); QoL, quality of life (physical functioning,role-physical, vitality, mental health); NT-proBNP, N-terminal B-type brain natriuretic peptide precursor; 6 MWD, 6-min walk distance; HbA1c, Glycated Hemoglobin A1c; KCCQ, kansas city cardiomyopathy questionnaire; sRAGE, a receptor for advanced glycation end products; CRP, C-reactive protein; TNF-α, Tumor Necrosis Factor-α; IL-6, interleukin-6; MDA, malondialdehyde; GSH-Px, glutathione peroxidase; SOD, superoxide dismutase; TCM, syndromes, Syndromes in Traditional Chinese Medicine; E/e′, Early left ventricular filling velocity/Early mitral annular diastolic velocity; SPPB, short physical performance battery (evaluates balance, walking speed, and sitting and standing time, with a total score of 0–12 points); LAVI, left atrial volume index.

### 4.1 Shexiang Baoxin pill

Shexiang Baoxin (SXBX) Pill, derived from an ancient Song Dynasty formula, is safe and effective as an adjunctive treatment for coronary heart disease, myocardial infarction, HF, and dyslipidemia. A recent study in patients with HFpEF showed primary measures (peak oxygen uptake (peak VO_2_) and anaerobic threshold (AT)), blood pressure, heart rate, blood oxygen level, the 6-min walking test (6 MWT), and sleep quality using the Pittsburgh Sleep Quality Index (PSQI) were improved in the integrated SXBX and GDMT group. No major adverse effects were reported (Liu et al, 2023). The results showed that SCBX Pill combined with exercise training was superior to exercise training alone in improving sleep quality and multiple dimensions of quality of life, especially physical functioning, role-physical, vitality, and mental health. SXBX featured distinct animal-human pharmacokinetics profiles, excellent clinical safety in the trials, and manageable CYP450/transporter-mediated drug interaction risks, but with no report about interaction with standard GDMT (Lu et al, 2018; Tao et al, 2016; Zhang et al, 2022).

### 4.2 Tinglu Yixin prescription

A clinical study evaluated Tinglu Yixin (TLYX) prescription in 108 HFpEF patients with type 2 diabetes over 12 weeks. Add-on botanical therapy significantly improved New York Heart Association (NYHA) functional class, improved diastolic function (LVEDD, LAD and E/e′ ratio, lowered biomarker (NT-proBNP) and biochemical indicator (HbA1c), and enhanced 6 MWT distance, quality of life, and TCM syndrome scores versus standard care alone. No serious adverse events or laboratory abnormalities occurred (Zhang and Wang, 2024). The findings supported TLYX as a safe, effective adjunctive therapy for HFpEF complicated by diabetes, addressing both cardiac remodeling and metabolic control. TLYX showed a promising usage in HFpEF with 168 active metabolites and safe clinical efficacy, but lacked absorption, distribution, metabolism, excretion (ADME) data and no report about interaction with standard GDMT.

### 4.3 San-jia-fu-mai decoction

A double-blind RCT evaluated San-jia-fu-mai Decoction (SJFM) as an adjunctive therapy in 110 HFpEF patients with AF, all receiving dapagliflozin-based optimal care. Over 12 weeks, SJFM significantly reduced NT-proBNP, soluble receptor for advanced glycation end-products (sRAGE), and E/e′, while improving KCCQ scores versus placebo, with superior effects emerging from week 8. At week 12, SJFM achieved a higher KCCQ grade improvement rate compared with the one in the placebo group. No serious adverse events occurred (Yang et al, 2025). The findings demonstrate that SJFM effectively alleviates cardiac wall stress, diastolic dysfunction, and systemic inflammation, conferring meaningful clinical benefits as a safe adjunctive therapy in this high-risk population (Yang et al, 2025). However, its pharmacokinetic profile remained unknown, studies on interaction with standard GDMT were entirely lacking, and long-term safety has not been systematically validated.

### 4.4 Jianxin granule

Jianxin Granule is commonly used for the treatment of HF. A clinical study has shown that Jianxin Granule could improve the TCM syndromes, reduce the level of systemic inflammation in elderly patients with HFpEF of the qi deficiency and blood stasis type by lowering serum levels of NT-proBNP and inflammatory factors (CRP, IL-1β, IL-6), and exhibited a good safety profile (Chen C. et al, 2022). A randomized controlled trail (RCT) study enrolled 98 patients with qi-deficiency and blood-stasis–type HFpEF, with 94 completing the analysis. After 8 weeks, Jianxin granules combined with standard therapy reduced CRP, TNF-α, IL-6 and malondialdehyde (MDA), but increased glutathione peroxidase (GSH-Px) compared with standard therapy alone, while SOD showed no significant between-group difference. No liver or kidney dysfunction, bleeding, purpura or rash was reported (Chen et al., 2024). However, the study assessed laboratory biomarkers rather than hospitalization or mortality outcomes. There were no reports about pharmacokinetic profile, long-term safety and interaction with standard GDMT.

### 4.5 QiShen YiQi dripping pills

Qishen Yiqi (QSYQ) Dripping Pills is widely used in China for the treatment of coronary heart disease with the TCM pattern of Qi deficiency and blood stasis (Lu et al, 2023). QSYQ as an adjunctive therapy could effectively improve exercise tolerance (6 MWD), physical function (short physical performance battery (SPPB)), and quality of life (KCCQ) in elderly patients with HFpEF, and might alleviate left atrial remodeling (reduction in left atrial volume index (LAVI)). No severe adverse effects reported. However, improvements in cardiac function indicators (LVEF) and biomarker (NT-proBNP) did not reach statistical significance (Li et al, 2026). The pharmacokinetic profile, long-term safety and interaction with standard GDMT have not been described.

### 4.6 Shenzhu Xinkang decoction

A clinical study evaluated Shenzhu Xinkang Decoction in 105 HFpEF patients over 16 weeks. Compared to standard care (dapagliflozin plus sacubitril/valsartan), the add-on prescriptions therapy significantly improved diastolic function indices (E, e′, E/A, E/e′, LAVI), reduced epicardial adipose tissue (EAT) thickness, NT-proBNP, and inflammatory markers including hs-CRP and TNF-α, while increasing interleukin-10 (IL-10). Cardiac remodeling markers (left ventricular end-diastolic diameter (LVEDD), left ventricular end-systolic diameter (LVESD), left atrial volume (LAV)) and TCM syndrome scores also improved more substantially in the treatment group, with superior overall response rates. No safety concerns emerged, confirming its good efficacy and tolerability (Yu et al, 2026). However, its pharmacokinetic profile remained unclear, studies on interaction with standard GDMT were entirely unknown, and long-term safety has not been systematically validated.

### 4.7 Clinical significance

Clinical observations from TCM prescriptions trials provide multifaceted insights into the pathogenesis and phenotype-specific therapeutic strategies for HFpEF. For example, SXBX was fit for dyslipidemia-phenotype, TLYX was favorable for diabetes-phenotype, SJFM was good for AF-phenotype and QSYQ was suitable for atrial myopathy-phenotype. Moreover, some metabolites (SAA, periplocin and limonin) and formula (Qidan formula) had the potential for hypertension-phenotype, limonin had the potential for dyslipidemia-phenotype and obesity-phenotype, as well as genipin and Qidan formula had the potential for diabetes-phenotype in preclinical research. HFpEF heterogeneity across different phenotypes challenges uniform treatment paradigms, aligning closely with TCM’s principle of pattern differentiation (Bian Zheng). Multi-metabolite prescriptions are tailored to simultaneously engage multiple phenotype-specific targets. At the mechanistic level, TCM prescriptions exert multi-metabolite and multi-target effects by simultaneously modulating inflammation, oxidative stress, and mitochondria dysfunction. At the therapeutic strategy level, these prescriptions have demonstrated improvements in exercise capacity, quality of life, symptom burden, and functional status, with a relative safety profile. Moreover, the “syndrome-method-formula” system of TCM prescriptions offers a clinical basis for developing phenotype-specific therapeutic strategies, forming a structural complement to the phenotype-oriented therapeutic concept of modern medicine.

### 4.8 Limitation of clinical evidence

Current HFpEF trials were methodologically limited: small samples, short follow-up (≤24 weeks), surrogate endpoints, nonstandard analyses, and precluding long-term prognostic assessment. This rendered the evidence a little low-quality and clinically relative unreliable. Besides, the studies we selected showed that TCM prescriptions could enhance therapeutic efficacy via serving an adjuvant to GDMT, but not the direct treatment for HFpEF. To enhance more credibility, future studies must adopt rigorous designs: multicenter, large-sample RCTs with adequate power, ≥1-year follow-up, and hard endpoints (e.g., cardiovascular death, HF rehospitalization) to systematically evaluate TCM prescriptions’ impact on long-term outcomes in HFpEF patients.

### 4.9 Other clinical studies

The conventional meta-analysis included 17 RCTs up to 2018 verified that some TCM prescriptions combined with GDMT brought obvious improvements but noted most trials had high bias risk (Wang et al, 2018). The Bayesian network meta-analysis incorporated 64 RCTs up to 2023 limited to oral TCM prescriptions identified that Qili Qiangxin Capsule and Shensong Yangxin Capsule delivered superior comprehensive benefits on cardiac biomarkers and exercise capacity (Guo et al, 2023). The narrative review collected 58 full-spectrum TCM prescriptions RCTs up to 2023 showed that various TCM prescriptions (such as Yiqi Yangyin Huoxue Decoction and Qi Li Qiangxin Capsules) could improve on cardiac function, biomarkers and exercise capacity (Fan et al, 2023).

## 5 Strengthen and limitation

Unlike prior reviews focusing on broad clinical efficacy and static target networks, an integrative translational framework for TCM in HFpEF was provided in this review. We discussed both metabolites and prescriptions from basic research to clinical research. By evaluating clinical evidence through phenotype-specific stratification, cross-model mechanics treating HFpEF potential and critically appraising clinical evidence grading, and incorporating pharmacokinetic and safety profiles, a clear roadmap connecting chemical quality control to phenotype-targeted clinical application was delineated.

However, there are also some limitations in this review. It is a narrative review rather than a systematic review. Search strategies combined core terms for “heart failure with preserved ejection fraction,” “inflammation,” “oxidation,” “mitochondria,” “Traditional Chinese Medicine/natural products” with specific metabolites and prescriptions, and “guideline-directed medical therapy.” 590 deduplicated records against strict eligibility criteria were included, excluding non-HFpEF studies, conference papers, and case reports. Ultimately, 105 studies (*in vitro*, *in vivo*, and clinical) were included. The data regarding TCM, GDMT, models, pathways, and outcomes were extracted and summarized using narrative approach due to methodological heterogeneity by three investigators. The cited literature consisted predominantly of studies reporting positive results, whilst those with negative or inconclusive findings might not been adequately included. Therefore, selection bias may be present and it influences the conclusion.

## 6 Conclusion and future perspectives

In this review, we focus on the pathophysiological mechanisms and pharmcological strategy for HFpEF, especially in TCM (Figure 4). Pharmacological actions were mechanistically paired with pathophysiological cascades, drugs modulated the defined disease pathways (such as inflammation, oxidation or mitochondrial dysfunction), establishing a possible cause-and-effect link between drug intervention and systemic restoration.

**FIGURE 4:**
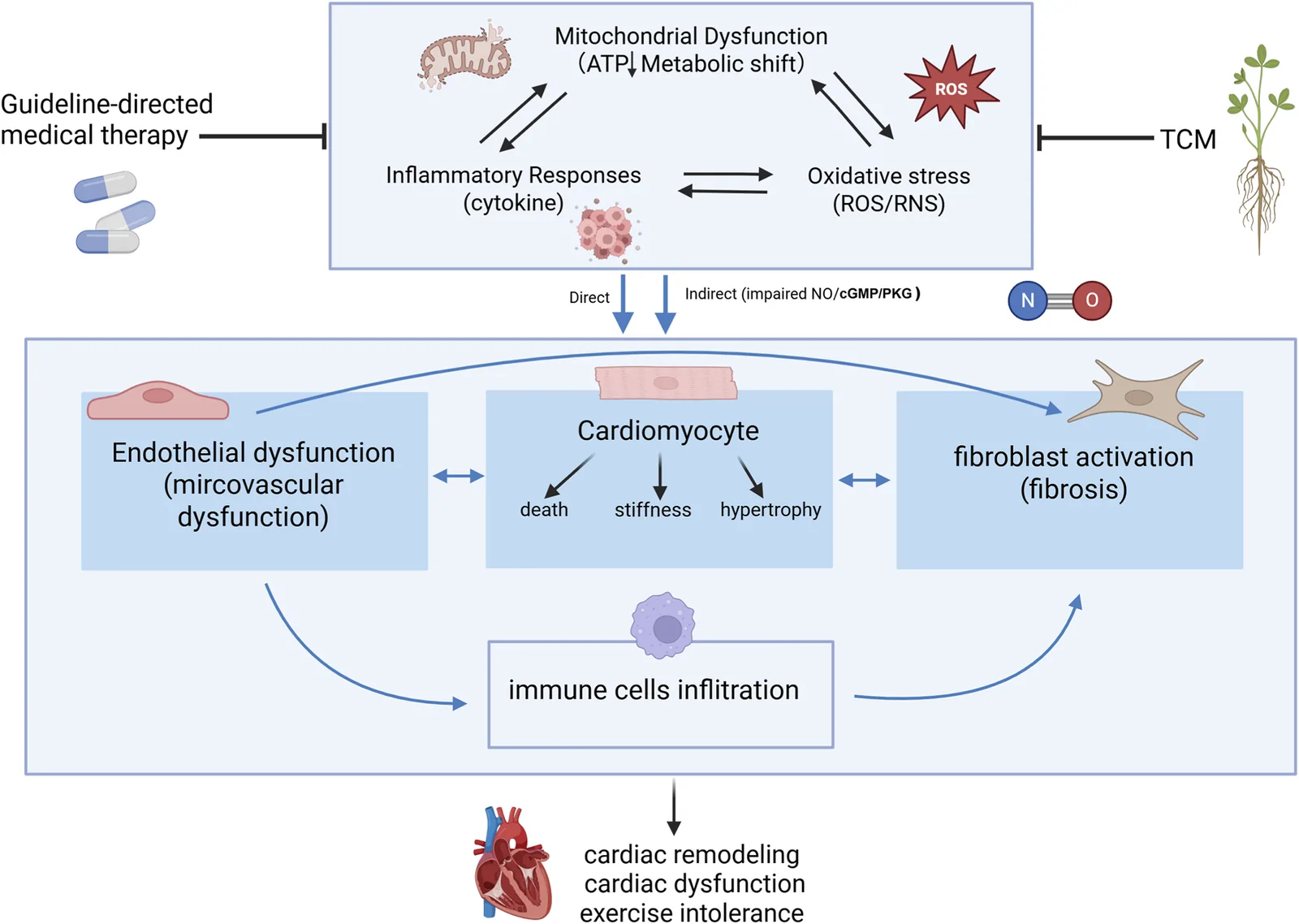
Pathophysiological mechanisms and pharmacological therapeutic strategy of Created with BioRender.com.

The regular medical treatments of HFpEF were SGLT2 inhibitors, MRAs, ARNI and GLP-1R agonists shown therapeutic prospects through some mechanisms. Many studies have shown that TCM metabolites and prescriptions could improve cardiac function and remodeling in HFpEF through multi-target and multi-pathway (anti-inflammation, anti-oxidation and improve mitochondrial dysfunction) in preliminary findings. Some TCM as adjuvant treatment could also significantly improve exercise tolerance and quality of life of patients with high safety in clinical research. These TCMs are often based on TCM theories such as “invigorating qi and activating blood circulation,” “nourishing Yin and warming Yang,” “resolving phlegm and clearing collaterals,” and embody the characteristics of holistic regulation and syndrome differentiation.

While multi-metabolite TCM offers broad, multi-target efficacy for HFpEF, rigorous safety profiling remains essential. Botanical drug-drug interactions need attention particularly, as some metabolites and prescriptions can induce or inhibit CYP450 enzymes and drug transporters, unpredictably altering the clearance of concurrent cardiovascular medications. Furthermore, risks of potential hepatic or renal toxicity underscore the need for strict dose standardization and quality control. Implementing comprehensive, long-term post-marketing pharmacovigilance is vital to monitor real-world toxicities and ensure TCM safe integration into modern clinical practice.

HFpEF is a highly heterogeneous syndrome, and its prevention and treatment strategies should pay more attention to accurate classification and individualized treatment. With the development of omics technology, artificial intelligence and imaging, it is expected to establish a fine classification system based on biomarkers, genetic background and clinical phenotype in the future, so as to achieve more precise intervention. Through in-depth exploration of its pathological mechanism, strengthening clinical transitional research, and promoting the usage of TCM, more effective and safer treatment options or adjuvant for HFpEF patients will be provided, and ultimately improve their long-term prognosis and quality of life.
